# Assessing Efficacy of Interleukin-1 Blockade in Patients with Alcoholic Hepatitis: A Comprehensive Systematic Review of Emerging Evidence

**DOI:** 10.3390/life15071106

**Published:** 2025-07-15

**Authors:** Shree Rath, Mary Girgis, Ishita Gupta, Anchit Chauhan, Zahir Ud Din, Hema Hotchandani, Ali Hasan, Raheel Ahmed, Raheel Qureshi

**Affiliations:** 1 All India Institute of Medical Sciences Bhubaneswar, Bhubaneswar 751019, India; shreerath4a@gmail.com; 2 Faculty of Medicine, Cairo University, Cairo 12613, Egypt; girgismary25@gmail.com; 3 St. Bernards Medical Center, Jonesboro, AR 72401, USA; igupta@sbrmc.org; 4 Department of Medicine, Maulana Azad Medical College, Delhi 110002, India; anchitchauhan645@gmail.com; 5 Department of Medicine, Khyber Medical College, Peshawar 25120, KPK, Pakistan; zaheerbnv@gmail.com; 6 SUNY Upstate Medical University, Syracuse, NY 13210, USA; hemahotchandani1113@gmail.com; 7 Imperial College London, London W12 0NN, UK; ali.hasan21@imperial.ac.uk; 8 National Heart and Lung Institute, Imperial College London, London SW3 6LY, UK; 9 Department of Gastroenterology, Queen Elizabeth Hospital, Gateshead NE9 6SX, UK; raheelqureshi@nhs.net

**Keywords:** alcoholic hepatitis, interleukin-1, prednisolone, canakinumab, anakinra

## Abstract

*Background and Objectives*: Alcoholic hepatitis (AH) is a growing public health concern with its rising incidence and its contribution to nearly half of all cirrhosis-related deaths in the United States. In this systematic review, we aimed to comprehensively evaluate the current evidence and trials on the use of anti-interleukin-1 (anti-IL-1) drugs in patients with AH, assessing their efficacy and adverse events compared to routinely prescribed drugs like corticosteroids. *Materials and Methods*: A comprehensive literature search was conducted across five databases to identify randomized controlled trials (RCTs) evaluating the role of anti-IL-1 agents like canakinumab and anakinra among patients diagnosed with AH. Data was extracted and pooled in the form of mean and standard deviation for continuous variables and event and total for dichotomous variables. *Results*: Three RCTs were included for quantitative synthesis, encompassing 307 patients. Using canakinumab, 58% of patients showed improvement in histology. Prednisolone was associated with higher 90-day survival (90% vs. 70%; hazard ratio for death = 0.34, 95% CI 0.14–0.83, *p* = 0.018) and transplant-free survival. Overall, a higher incidence of acute kidney injury and new-onset cardiac disorders was noted in the anti-IL-1 arm when compared to placebo. *Conclusions*: This study concludes the lack of efficacy of anti-IL-1 agents in causing improvement in patient outcomes when compared to standard therapies. A higher incidence of adverse events was also noted in the anti-IL-1 arm. These results emphasize the need for future clinical trials to evaluate the use of anti-IL-1 agents in AH objectively.

## 1. Introduction

Alcohol has always been causatively linked with liver disease in statistically and clinically significant data [1], causing various stages of liver disease (fat deposition, inflammation, end-stage liver disease). Alcoholic hepatitis is usually the acute onset of symptomatic hepatitis due to alcohol [2]. Consumption of more than 100 g of alcohol per day for more than a decade has been widely seen in patients with alcoholic liver disease, although an exact amount has not yet been established [3]. Commonly seen in 40 to 50-year-old individuals [4], the burden of alcoholic liver disease is an ever-growing concern in the modern world, accounting for 5.9% of all deaths worldwide in 2018 [5]. In the United States alone, alcohol-associated liver disease led to over 12.53 deaths per 100,000 population in 2022, showing a rise of 8.94% annually [6]. We have observed a significant rise in alcoholic liver disease-related hospitalization [7] and an even greater increase in both hospitalization and referrals for liver transplant during the COVID-19 pandemic [8,9].

Clinically, a moderate elevation in aspartate aminotransferase (AST) and alanine aminotransferase (ALT) and a two-to-one ratio between AST and ALT is classic for alcohol-related liver injury [10,11,12] and patients can present with a spectrum of clinical disease ranging from steatohepatitis to cirrhosis [6] with the clinical course being determined by whether or not abstinence from alcohol is followed by the patient [13,14]. Current guidelines on management of acute alcoholic hepatitis include supportive care like nutrition support, sepsis prevention, ulcer prophylaxis, management of alcohol withdrawal, management of hepatic encephalopathy [6] and the use of pharmacological agents like steroids based on the Lille score [15,16] with liver transplantation being the definitive step for end-stage liver disease [6]. Although interleukin-1 (IL-1) inhibitors like anakinra, rilonacept and canakinumab do not form a part of guideline-based clinical treatment, studies have shown promising results in alcoholic hepatitis [17,18].

In 2011, a study conducted on mice suggested that alcohol affects the Hypoxia Inducible Factor 1 (HIF) and other fatty acid metabolism regulators, eventually resulting in pathological fat deposition in hepatocytes [19]. This in turn leads to the generation of a pro-inflammatory response due to the recruitment of cells like macrophages, monocytes, and neutrophilic leucocytes [20,21]. IL-1 is a proinflammatory mediator for this immune defense. It skews the differentiation of T cells toward proinflammatory T helper 17 (Th17) cells, while helping CD4+ and CD8+ T cells function [22,23]. IL-1 has been found to be high in the serum of patients with alcoholic hepatitis [24]. Blocking and inhibiting the effect of this highly potent pro-inflammatory cytokine by commercially available agents like anakinra, rilonacept and canakinumab may affect alcoholic hepatitis [25].

**Figure 1:**
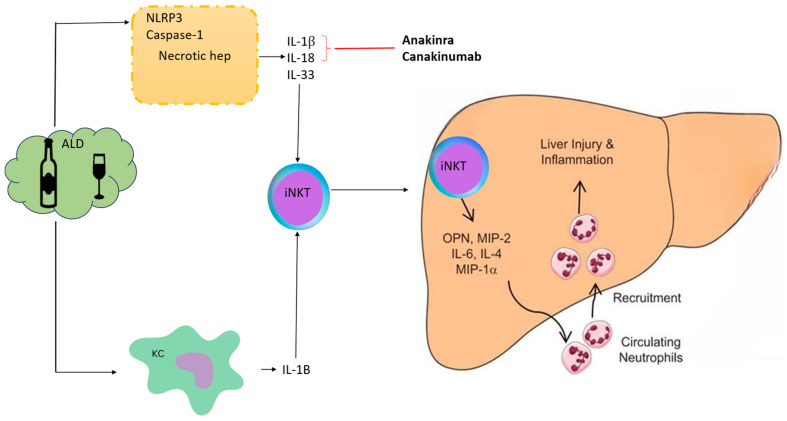
Mechanism of action of anti-IL-1 drugs in alcoholic hepatitis. Alcohol induces injury to Kupffer cells and hepatocytes by activating the inflammasome NLRP3-caspase 1, leading to the production of IL-1β from Kupffer cells and hepatocytes, as well as IL-18 & IL-33 from hepatocytes, in ALD. IL-1β and IL-18 recruit and activate iNKT cells that, in turn, induce neutrophil afflux and hepatic injury. IL-33 released by damaged and necrotic hepatocytes exacerbates iNKT cells and neutrophil recruitment and activation. IL-1 inhibitors like Anakinra and Canakinumab block these cytokines. ALD, alcoholic liver disease; KC, Kupffer cells; iNKT, invariant natural killer T-cells; Osteopontin (OPN), also known as early T lymphocyte activation; MIP: Macrophage Inflammatory Proteins.

Figure 1 provides a brief mechanism of action of IL-1 drugs. Alcohol induces injury to Kupffer cells and hepatocytes by activating the inflammasome NLRP3-caspase 1 and production of IL-1β from Kupffer cells and hepatocytes & IL-18 & IL-33 from hepatocytes in ALD. IL-1β and IL-18 recruit and activate iNKT cells that, in turn, induce neutrophil afflux and hepatic injury. IL-33 released by damaged and necrotic hepatocytes exacerbates iNKT cells and neutrophil recruitment and activation. IL-1 inhibitors like anakinra and canakinumab block these cytokines.

Petrasek et al. conducted a study in mice and concluded that IL-1 inhibitors can attenuate liver injury in alcoholic liver disease [26]. Similar results were seen in a 2017 study conducted in the United States [27]. An RCT conducted by Gawrieh et al. in 2024 [28] compared anakinra plus zinc with steroids. Although the mortality benefit of this study was not statistically significant, it did demonstrate a treatment effect in favor of the combination group [28]. Another randomized controlled trial has recently been started by the Imperial College, London (ISAIAH), looking into the effects of canakinumab in alcoholic hepatitis [29].

While the evidence suggests that IL-1 inhibitors can be of extensive use in alcoholic hepatitis, our knowledge in this arena is still far from complete. In recent years, alcoholic liver disease has been the focus of health authorities around the world as it not only increases the health and mortality burden but also the economic burden [6]. As the literature around the use of IL-1 inhibitors is evolving at a rapid rate, we conducted a global systematic review to appraise the existing literature on the use of IL-1 inhibitors like anakinra, rilonacept and canakinumab in alcoholic hepatitis in terms of clinical benefits, outcomes and adverse effects. Organizing summaries of the available clinical evidence regarding safety and effectiveness from published literature through a systematic review can provide a synopsis, which in turn may help plan large-scale randomized controlled trials in the future and guide clinicians on the use of these agents in their routine practice.

## 2. Materials and Methods

### 2.1. Search Method and Strategy

A literature search was conducted from inception to September 2024 for articles on IL-1 inhibitors for alcoholic liver disease following PRISMA guidelines [30]. Primary databases used for the search were PubMed, Embase, Scopus, Web of Science and Cochrane. The detailed search strategy is summarized in Supplementary Table S1. The keywords used for the search strategy were interleukin-1 inhibitors, IL-1 inhibitors, anakinra, rilonacept, canakinumab, alcoholic hepatitis, steatohepatitis, fatty liver and their combinations. After a thorough search, full-length articles meeting the inclusion criteria were evaluated. Subsequently, a manual search for the references of the articles included was conducted. The study was prospectively registered on PROSPERO (CRD420251060482).

### 2.2. Data Screening and Eligibility

Articles were screened for eligibility based on the following inclusion criteria; (1) Population: Patients suffering from any stage of alcohol-induced liver damage, including steatohepatitis, hepatitis, fatty liver, etc.; (2) Intervention: use of IL-1 inhibitor like anakinra, rilonacept, canakinumab; (3) Control: Use of conventional therapies for alcoholic liver disease; (4) Study designs: randomized and non-randomized clinical trials, observational studies, cross sectional, case–control, cohort studies.

Our exclusion criteria were as follows:

1. Pregnant patients.
2. Non-peer-reviewed articles, commentaries, news reports or studies published as abstracts only.

Out of 3387 published studies, 3 studies met the eligibility criteria and were included in the final review. Each article was reviewed by two authors independently (AC, ZD). The disagreements were discussed and resolved by reaching a common consensus.

### 2.3. Data Collection and Analysis

Subsequently, the data was collected and tabulated using Microsoft Excel. The included data was checked for accuracy by all authors. The primary and secondary outcomes assessed were

1. 90-day survival.
2. Transplant-free survival.
3. Adverse events.

### 2.4. Quality Assessment

Quality assessment of included studies was conducted using Risk of Bias-II (ROB2). ROB2 investigates the risk of bias according to five domains (randomization, deviations from intended interventions, missing outcome data, outcome measurement and selection of the reported result). Any conflict was handled through discussion to make a final decision [31].

## 3. Results

### 3.1. Baseline Characteristics

**Figure 2:**
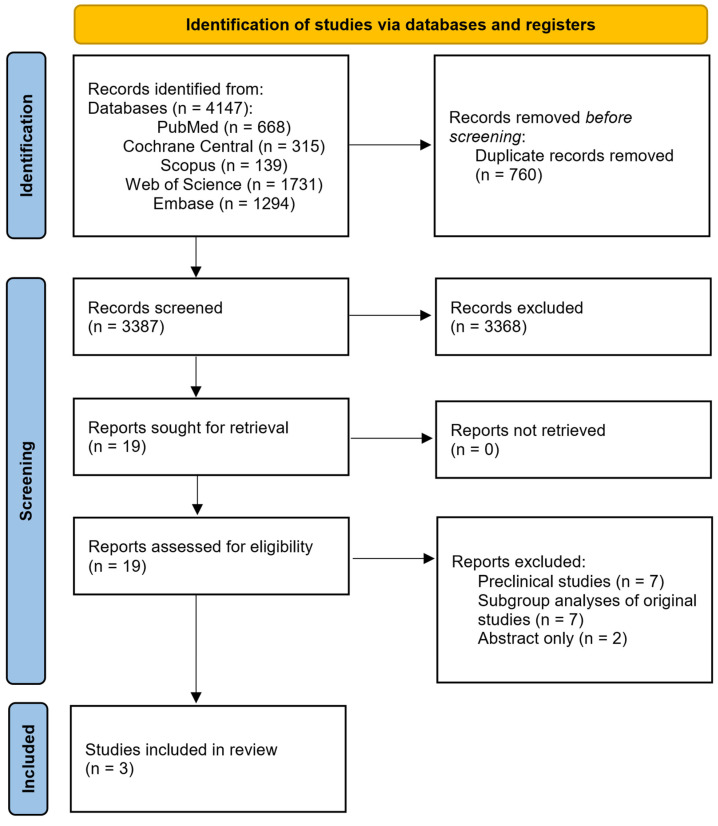
PRISMA chart of included studies.

After performing a thorough search through the available scientific literature on alcoholic hepatitis and the use of IL-1 inhibitors, three studies met the inclusion criteria and were included in the final qualitative analysis [28,29,32] (Figure 2).

The included trials enrolled adult patients with severe alcoholic hepatitis, defined by clinical criteria such as a Model for End-Stage Liver Disease (MELD) score ≥ 20 or a Maddrey Discriminant Function (mDF) ≥ 32. All studies required recent or ongoing heavy alcohol consumption, with thresholds ranging from >50 g/day for over 6 months [32] to ≥80 g/day for men and ≥60 g/day for women [29]. Common exclusion criteria across the trials included active infections (including COVID-19), renal failure or acute kidney injury, gastrointestinal bleeding, pancreatitis, significant comorbidities (cardiac or neurologic disease), malignancy, HIV or evidence of other chronic liver diseases. Additional exclusions were prior corticosteroid or immunosuppressive use, pregnancy and jaundice lasting more than 3 months [29].

All the studies included were randomized controlled trials. Two of the studies [28,32] were conducted in the USA, and one in the UK [29]. A total of 307 participants were included. The mean age was 46.6 and 45.6 years in the intervention and control groups, respectively, and there were 63.5% male participants in the intervention group and 53.4% in the control group. The mean MELD scores of all participants in all three studies were 23.5 and 24 in the intervention and control groups, respectively (Table 1).

### 3.2. Quality Assessment

**Figure 3:**
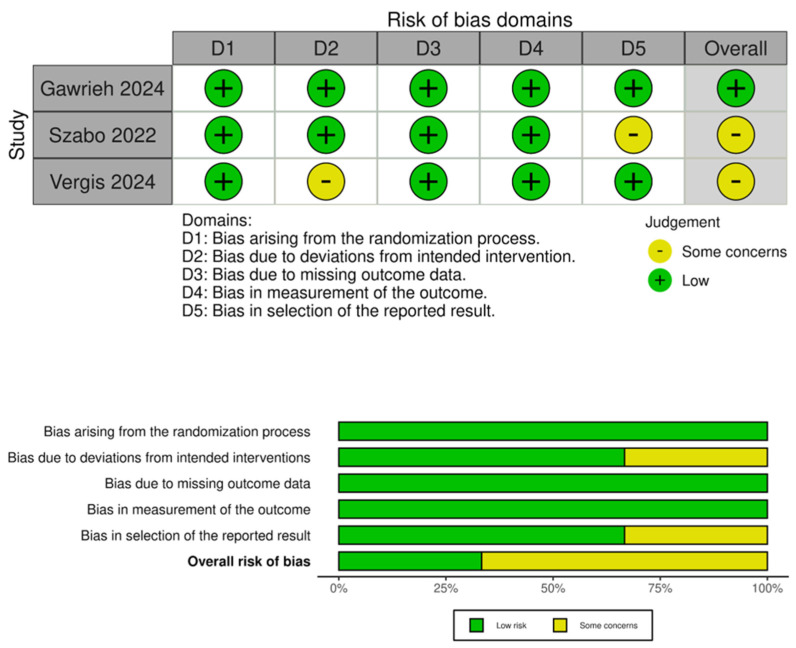
Quality assessment of included trials [28,29,32].

Quality assessment was conducted using RoB-2. One trial had a low risk of bias, while two had some concerns due to confounding in intervention and reporting of outcomes. Further details are provided in Figure 3.

### 3.3. Drugs Used in Both Intervention and Control Groups

Two studies compared zinc with either a placebo or with prednisolone [28,32], whereas one study compared canakinumab with a placebo [29]. Gawrieh et al. [28] used subcutaneous Anakinra (100 mg once daily for 14 days) in combination with oral zinc (220 mg once daily for 90 days) and compared this with oral prednisolone (40 mg once daily for 30 days) [31]. Another study conducted by Szabo in 2022 compared subcutaneous anakinra (100 mg daily for 14 days) + oral pentoxifylline (400 mg three times daily for 28 days) + oral zinc sulfate (220 mg for 180 days) with oral prednisolone (32 mg daily for 28 days) [32]. The third trial, which was conducted in the UK, compared canakinumab with a placebo [29]. In this study, an IV infusion of canakinumab was compared with dextrose solution. In the same study, participants also received Bactrim prophylaxis for the first 14 days [29].

### 3.4. Outcomes

In the study that compared canakinumab with a placebo, 58% of patients in the canakinumab group showed improvement in histology, whereas this was seen in only 42% of patients in the placebo group. These results were not statistically significant. It also did not translate into clinical benefits. However, mortality and risk of adverse effects were comparable in both groups [29]. Gawreih et al. [28] trial showed that statistically significant results were seen with prednisone being associated with higher 90-day survival (90% vs. 70%; hazard ratio for death = 0.34, 95% CI 0.14–0.83, *p* = 0.018) and transplant-free survival (88% vs. 64%; hazard ratio for transplant or death = 0.30, 95% CI 0.13–0.69, *p* = 0.004) when compared with anakinra + zinc. The study also noted that anakinra + zinc caused more AKI when compared with placebo [28]. Similar results were seen in the trial conducted by Szabo et al. [32]; nonsignificant results were noted in the Kaplan–Meier survival estimate at 180 days, whereas survival at 28 days was similar between both the intervention and control groups.

### 3.5. Adverse Effects

A total of 112 adverse effects in the interventional group and 92 in the control group were noted among all three trials. Acute kidney injury was seen the most, with 44 participants in the intervention group and 30 participants in the control group experiencing it. Septic shock was more frequently seen in the control group (10 participants), whereas only six patients in the control group were noted to experience septic shock. Opposite effects were seen in the overall infection rates; 35 patients in the intervention group and 29 in the control group were noted to have one or more infections. Upper GI hemorrhage was seen in four participants from each group. Szabo et al.’s study found that seven participants in the interventional group and two in the control group were found to have a cardiac disorder during treatment [32]. Szabo et al. also found that three patients in the intervention group and five in the control group experienced an episode of acute cholecystitis during the treatment [32]. Ascites was seen in one patient in the control group of the study by Vergis et al. [29]. Table 2 provides a summary of all adverse events reported across the included trials.

## 4. Discussion

In our review, three randomized controlled trials (RCTs) were included, with 307 participants, mainly from the USA and the UK. Among the studies, comparable baseline characteristics, namely age (~46 years), sex distribution (~60% male) and MELD scores (~23–24), reduce confounding factors and make the results more reliable. However, the majority of participants being from the UK and USA reduces the generalizability [28,29,32].

A study comparing canakinumab with a placebo found that 58% of the canakinumab group showed histological improvement, compared to 42% in the placebo group, but this did not lead to significant clinical benefits or reduced mortality [29]. This suggests that histological improvement may not always predict better clinical outcomes, like survival. Longer follow-ups and larger sample sizes are needed to confirm any meaningful effects.

To understand this apparent paradox, we should focus on the complex, multifactorial pathogenesis of severe alcoholic hepatitis, which extends far beyond simple inflammatory cytokine dysregulation. While IL-1β plays a central role in hepatic inflammation and is significantly elevated in AH patients (almost 10-fold) [33], the pathophysiology of alcoholic hepatitis involves an intricate network of interconnected mechanisms that cannot be addressed by targeting a single inflammatory pathway [34]. These include oxidative stress from CYP2E1-mediated alcohol metabolism, which generates reactive oxygen species and depletes antioxidant defenses [35]. Mitochondrial dysfunction occurs through ROS-induced structural and functional damage, leading to impaired cellular energy production and enhanced apoptotic signaling [36]. The gut–liver axis contributes through alcohol-induced intestinal barrier dysfunction, allowing bacterial translocation and endotoxin-mediated Kupffer cell activation [37]. Additionally, impaired hepatocyte regeneration, dysregulated lipid metabolism, acetaldehyde toxicity and formation of protein–acetaldehyde adducts all contribute to ongoing liver injury [34]. This suggests that histological improvement may not always predict better clinical outcomes, like survival.

The trial by Gawrieh et al., which compared the use of anakinra combined with zinc to prednisone in treating severe alcoholic hepatitis, showed that prednisone was more effective, with a 90-day survival rate of 90% compared to 70% in the anakinra plus zinc group [28]. Prednisone also demonstrated better transplant-free survival rates, with 88% of patients surviving without a transplant, compared to 64% in the other group possibly because anakinra targets the inflammatory pathway, so it may not adequately address other critical pathophysiological mechanisms, such as oxidative stress and liver regeneration, which are better managed by corticosteroids [38]. Although zinc may provide additional benefits, these were not enough to match the well-established effects of prednisone in this condition [39].

The Szabo et al. [32] study showed no significant difference in 180-day survival between anakinra + zinc and prednisolone. There was little difference in survival at 180 days compared to that at 90 days. This suggests that using 90-day survival as an endpoint might be more appropriate for future phase II or III trials in alcoholic hepatitis, as has been recommended in recent research [32,40]. The short-term survival benefit seen with anakinra may be due to its ability to reduce acute inflammation [41]. However, its lack of long-term impact indicates that blocking IL-1 alone may not be enough to prevent ongoing liver damage or complications like infections [42].

IL-1 inhibitors showed higher rates of adverse effects, particularly acute kidney injury (AKI) (44 vs. 30 participants), and overall infections (35 vs. 29 participants). IL-1 inhibitors, while reducing inflammation, may impair other immune defenses, predisposing patients to infections [43]. AKI, a significant concern, could result from compounded effects of systemic inflammation, pre-existing liver dysfunction and potential nephrotoxicity of the treatment itself [44].

Interestingly, septic shock occurred slightly more often in the control group, which may indicate that the anti-inflammatory effect of IL-1 inhibitors provides some protection against severe inflammation that leads to such complications.

Histological changes with canakinumab showed only some improvement, suggesting that, while it has anti-inflammatory effects, these are not strong enough to result in significant clinical benefits. Similarly, biochemical markers like MELD scores did not show any significant improvement in the studies.

The findings highlight important points about the use of IL-1 inhibitors in severe alcoholic hepatitis. While these drugs can reduce inflammation, they are less effective than corticosteroids in improving survival rates and reducing the need for liver transplants. Moreover, the safety of IL-1 inhibitors is a concern due to the higher risk of acute kidney injury (AKI) and infections. This means that patients need to be carefully selected and closely monitored during treatment. Although these drugs show promising effects on liver tissue changes under the microscope, such improvements alone are not enough to support their routine use in clinical practice unless they also lead to better survival or quality of life for patients, for which more evidence is required, as stated.

Our review has several limitations. First, only three RCTs were included, with a total sample size of just 307 participants. This limited number of studies significantly restricts the statistical power to detect meaningful differences in outcomes. Moreover, the small sample size makes it difficult to assess rare but clinically important adverse events or to draw firm conclusions about treatment efficacy across diverse patient subgroups.

The limited evidence base also means that our findings may be disproportionately influenced by the design or outcomes of individual trials. Given the small number of studies, meta-analytic techniques were not feasible, and conclusions are necessarily narrative in nature, which further limits the robustness of the evidence.

Another important limitation is the geographic concentration of the included studies, all of which were conducted in the United States and the United Kingdom. This regional focus may limit the global applicability of the findings, as outcomes in alcoholic hepatitis can vary significantly depending on factors such as genetic background, alcohol consumption patterns, nutritional status, comorbidities and access to healthcare services. For instance, populations in low- and middle-income countries (LMICs) may experience different disease trajectories and treatment responses due to variations in baseline liver disease severity, delays in presentation and differences in supportive care. Additionally, some heterogeneity in the IL-1 inhibitors used, such as anakinra versus canakinumab or their combinations with zinc, makes it difficult to directly compare outcomes. The interventions varied among the included trials, including different IL-1 inhibitors (anakinra and canakinumab), the use of adjunct therapy (zinc) and different control groups (placebo vs. prednisone). This variability makes it impossible to pool the data quantitatively and draw a single, unified conclusion about the drug class as a whole. Furthermore, lack of long-term follow-up data restricts our ability to assess sustained efficacy or late-onset adverse effects.

To address these gaps, future research should include larger, multicenter trials across diverse populations, ideally through international collaborations. Such studies would enhance statistical power and improve the representativeness of findings. Collaborative research efforts may also allow harmonization of study protocols and outcome measures, facilitating pooled analyses and meta-research.

In interpreting the findings of this review, it is also essential to critically evaluate whether trial design factors, patient selection criteria or choice of outcome measures may have contributed to the observed lack of significant benefit from IL-1 inhibitors in severe alcoholic hepatitis. The lack of stratified randomization based on baseline inflammatory markers or liver function may have obscured potential benefits in responsive subgroups.

Second, the comparators and co-interventions used in the trials may have influenced outcomes. For example, comparing IL-1 inhibitors to prednisolone, which is already established as standard of care in severe alcoholic hepatitis, sets a high benchmark, possibly underestimating the clinical utility of IL-1 blockade in settings where corticosteroids are contraindicated. In some studies, the addition of zinc to anakinra introduced a layer of complexity that may have influenced outcomes independently or through drug–nutrient interactions.

Third, the selection of primary endpoints warrants scrutiny. Most trials focused on short-term survival (90 or 180 days), which, while clinically relevant, may not fully capture long-term liver-related outcomes such as progression to cirrhosis, development of hepatocellular carcinoma or quality of life. Moreover, the reliance on biochemical markers and histological improvement as surrogate endpoints, which failed to correlate with survival benefits, raises concerns about their appropriateness in guiding treatment decisions.

In our included studies, the MELD score was commonly used to assess disease severity and prognosis. While MELD is a well-validated tool, particularly in the context of liver transplant prioritization, it is important to acknowledge the role of Maddrey’s Discriminant Function (mDF) and the Lille score in the clinical management of alcoholic hepatitis. mDF remains one of the earliest and most widely used prognostic scores, with values ≥32 typically indicating severe disease and prompting consideration of corticosteroid therapy [45].

Moreover, the Lille score, calculated after 7 days of corticosteroid therapy, provides a dynamic assessment of treatment response and is highly predictive of short-term survival. A Lille score > 0.45 generally suggests poor response to corticosteroids and may justify early discontinuation or redirection of therapy [46]. Although the Lille score was not prominently reported or analyzed in the included IL-1 inhibitor trials, its integration in future studies may provide valuable insights into early treatment response and help guide adaptive therapeutic strategies, especially in head-to-head comparisons with corticosteroids.

Additionally, the broader context of alcohol-related liver disease (ALD) must be considered. Alcohol consumption, even at modest levels, has been implicated not only in cirrhosis but also in the pathogenesis of multiple malignancies, including hepatocellular carcinoma (HCC), oropharyngeal cancers and colorectal cancers [47,48]. Therefore, future trials should consider alcohol use itself as a biological variable, examining how IL-1 inhibition interacts with the oncogenic and immunomodulatory effects of chronic alcohol exposure. Integrating biomarkers of inflammation, fibrosis and carcinogenesis may offer insights into which subgroups derive benefit from IL-1 blockade.

In addition to monotherapy strategies, combinatorial regimens involving IL-1 inhibitors with antioxidants to target oxidative stress, gut microbiota-modulating agents, given the known gut–liver axis in ALD, or anti-fibrotic drugs should be explored. Finally, identification of predictive biomarkers, including cytokine profiles or genetic predispositions, could help guide personalized therapy for patients with severe alcoholic hepatitis.

## 5. Conclusions

IL-1 inhibitors show promise as a treatment for alcoholic hepatitis, but they cannot yet replace corticosteroids like prednisone, which remains more effective in improving short-term and medium-term survival. While IL-1 inhibitors have shown encouraging results, such as better liver tissue changes and manageable side effects, more research is needed to confirm the findings. Larger, well-designed studies with diverse patient groups are required to better understand their role in treating alcoholic hepatitis.

## Figures and Tables

**Table 1:** Summary of included studies.

Study	Location	Study Design/Phase	Total Sample Size	Duration	Intervention	Regimen	Route	Control	Regimen	Route
Gawrieh et al. [28]	USA	phase IIb, double-blind, multicenter, randomized clinical trial	147 (73 + 74)	June 2020 to March 2022	Anakinra plus Zinc (A + Z)	daily anakinra 100 mg for 14 days daily zinc sulfate 220 mg orally for 90 days	A: subcutaneously Z: orally	Prednisone (PRED)	40 mg daily for 30 days	oral
Szabo et al. [32]	USA	Phase ll–lll, multicenter, randomized, double-blind trial	103 (53 + 50)	September 2013 to March 2018	Anakinra & Pentoxifylline & Zinc Sulfate (A + P + Z)	anakinra 100 mg daily for 14 days, pentoxifylline 400 mg three times daily for 28 day, zinc sulfate 220 mg for 180 days	A: subcutaneous; P: oral; Z: oral	methylprednisolone	32 mg daily for 28 days	Oral
Vergis et al. [29]	UK	randomized, double-blind, placebo-controlled, multicenter, phase ll exploratory trial	57 (29 + 28)	January 2019 to October 2020	Canakinumab	Canakinumab in 100 mL 5% dextrose solution	intravenous infusion	5% dextrose	100 mL of 5% dextrose solution only	intravenous

**Table 2:** Major adverse events across included trials. NR, not reported.

Study	Treatment Arm	Sample Size	Adverse Events								
			Acute Kidney Injury	Sepsis/Septic Shock	Upper Gastrointestinal Hemorrhage	Overall Infections	Fungal Infections	Cardiac Disorders	Abdominal Pain	Hematemesis	Acute Cholecystitis
Gawrieh et al. [28]	Anakinra & Zinc	74	33	5	NR	23	0	NR	NR	NR	NR
Gawrieh et al. [28]	Prednisolone	73	16	5	NR	20	1	NR	NR	NR	NR
Szabo et al. [32]	Anakinra & Pentoxifylline & Zinc Sulfate	53	10	NR	4	12	0	7	4	9	3
Szabo et al. [32]	Methylprednosolone	50	13	NR	4	9	5	2	8	3	5
Vergis et al. [29]	Canakinumab	28	1	1	NR	NR	NR	NR	NR	NR	NR
Vergis et al. [29]	Placebo	27	1	5	NR	NR	NR	NR	NR	NR	NR

## Data Availability

Data available in the manuscript and its Supplementary Files.

## Supplementary Materials

The following supporting information can be downloaded at: https://www.mdpi.com/article/10.3390/life15071106/s1, Table S1. Search strategy across included studies. Date of search: 7 September 2024.

## Abbreviations

The following abbreviations are used in this manuscript:

ALD	Alcoholic Liver Disease
IL-1	Interleukin 1
