# Supplementary material for: Assessing Efficacy of Interleukin-1 Blockade in Patients with Alcoholic Hepatitis: A Comprehensive Systematic Review of Emerging Evidence
Source: Life (Basel). 2025 Jul 15;15(7):1106. doi: 10.3390/life15071106 (PMC12299571; doi:10.3390/life15071106)
Supplement: Supplementary file 1 [file life-15-01106-s001.zip › life-3720574-supplementary.pdf]

Table S1. Search strategy across included studies. Date of search: 7 September 2024.

Database	Search String	Results
PubMed	(alcoholic liver OR alcohol-induced hepatitis OR alcoholic steatohepatitis OR fatty liver, alcoholic OR hepatitis, alcoholic OR liver cirrhosis, alcoholic OR alcoholic liver disease OR alcoholism OR alcohol-related liver disease OR alcohol-associated liver disease OR alcohol-related hepatitis OR alcohol* hepatitis OR alcoh* liver disease) AND (IL-1 OR IL1 OR anakinra OR rilonacept OR canakinumab OR "IL-1 antagonist" OR "IL-1 blocker" OR "IL-1 inhibitor" OR "interleukin 1")	668
Web of Science	(alcoholic liver OR alcohol-induced hepatitis OR alcoholic steatohepatitis OR fatty liver, alcoholic OR hepatitis, alcoholic OR liver cirrhosis, alcoholic OR alcoholic liver disease OR alcoholism OR alcohol-related liver disease OR alcohol-associated liver disease OR alcohol-related hepatitis OR alcohol* hepatitis OR alcoh* liver disease) AND (IL-1 OR IL1 OR anakinra OR rilonacept OR canakinumab OR IL-1 antagonist OR IL-1 blocker OR IL-1 inhibitor OR interleukin 1)	1731
Cochrane Central	(alcoholic liver OR alcohol-induced hepatitis OR alcoholic steatohepatitis OR fatty liver, alcoholic OR hepatitis, alcoholic OR liver cirrhosis, alcoholic OR alcoholic liver disease OR alcoholism OR alcohol-related liver disease OR alcohol-associated liver disease OR alcohol-related hepatitis OR alcohol* hepatitis OR alcoh* liver disease) AND (IL-1 OR IL1 OR anakinra OR rilonacept OR canakinumab OR IL-1 antagonist OR IL-1 blocker OR IL-1 inhibitor OR interleukin 1)	315
Scopus	TITLE-ABS-KEY((alcoholic liver OR alcohol-induced hepatitis OR alcoholic steatohepatitis OR fatty liver, alcoholic OR hepatitis, alcoholic OR liver cirrhosis, alcoholic OR alcoholic liver disease OR alcoholism OR alcohol-related liver disease OR alcohol-associated liver disease OR alcohol-related hepatitis OR alcohol* hepatitis OR alcoh* liver disease) AND (IL-1 OR IL1 OR anakinra OR rilonacept OR canakinumab OR "IL-1 antagonist" OR "IL-1 blocker" OR "IL-1 inhibitor" OR "interleukin 1"))	139
Embase	('alcoholic liver'/exp OR 'alcoholic liver' OR (('alcoholic'/exp OR alcoholic) AND ('liver'/exp OR liver)) OR 'alcohol-induced hepatitis' OR ('alcohol induced' AND ('hepatitis'/exp OR hepatitis)) OR 'alcoholic steatohepatitis'/exp OR 'alcoholic steatohepatitis' OR (('alcoholic'/exp OR alcoholic) AND ('steatohepatitis'/exp OR steatohepatitis)) OR 'fatty liver, alcoholic'/exp OR 'fatty liver, alcoholic' OR (fatty AND ('liver,'/exp OR liver,) AND ('alcoholic'/exp OR alcoholic)) OR 'hepatitis, alcoholic'/exp OR 'hepatitis, alcoholic' OR (('hepatitis,'/exp OR hepatitis,) AND ('alcoholic'/exp OR alcoholic)) OR 'liver cirrhosis, alcoholic'/exp OR 'liver cirrhosis, alcoholic' OR (('liver'/exp OR liver) AND ('cirrhosis,'/exp OR	1294

	cirrhosis,) AND ('alcoholic'/exp OR alcoholic)) OR 'alcoholic liver disease'/exp OR 'alcoholic liver disease' OR (('alcoholic'/exp OR alcoholic) AND ('liver'/exp OR liver) AND ('disease'/exp OR disease)) OR 'alcoholism'/exp OR alcoholism OR 'alcohol-related liver disease' OR ('alcohol related' AND ('liver'/exp OR liver) AND ('disease'/exp OR disease)) OR 'alcohol-associated liver disease' OR ('alcohol associated' AND ('liver'/exp OR liver) AND ('disease'/exp OR disease)) OR 'alcohol-related hepatitis' OR ('alcohol related' AND ('hepatitis'/exp OR hepatitis)) OR 'alcohol* hepatitis' OR (alcohol* AND ('hepatitis'/exp OR hepatitis)) OR 'alcoh* liver disease' OR (alcoh* AND ('liver'/exp OR liver) AND ('disease'/exp OR disease))) AND ('il 1'/exp OR 'il 1' OR il1 OR 'anakinra'/exp OR anakinra OR 'rilonacept'/exp OR rilonacept OR 'canakinumab'/exp OR canakinumab OR 'il-1 antagonist' OR 'il-1 blocker' OR 'il-1 inhibitor')	
